# The use of thermal imaging in evaluating musculoskeletal disorders in dentists

**DOI:** 10.25122/jml-2019-0017

**Published:** 2019

**Authors:** Mioara-Raluca Cosoroaba, Liviu Cirin, Mirella Dorina Anghel, Cristina Ioana Talpos-Niculescu, Veronica Argesanu, Andrei Zoldan Farkas, Meda Lavinia Negrutiu

**Affiliations:** 1. “Victor Babeş” University of Medicine and Pharmacy Timişoara, Romania; 2. Polytechnic University of Timişoara, Romania

**Keywords:** dentistry, ergonomics, musculoskeletal disorders, posture, thermography

## Abstract

Musculoskeletal disorders (MSDs) caused by incorrect working positions among dentists is a serious health issue and one that leads to decreased productivity and quality of life. Muscle activity and strain is correlated with higher surface temperatures due to increased metabolic activity [2]. The main objective of this study is to evaluate, using thermal imaging, the muscular strain experienced by oral healthcare professionals during work depending on their position, and to assess whether periodic stretching exercises have an impact on preventing MSDs. The study included four subjects and used thermal imaging to evaluate the heat pattern produced by muscle strain in two different states, one while working in incorrect postures and the other after performing stretching exercises. We used a FLIRB200 thermal imaging camera to measure skin surface temperature changes of the underlining muscles in the cervical, right arm triceps, and lumbar areas. According to the imaging, all four subjects recorded a drop in temperature in evaluated muscle regions after performing stretching exercises, corresponding to a decrease in muscle strain. Thermal imaging can be effectively used to evaluate muscle strain and MSDs. Stretching exercises could be viewed as effective preventive measures to avoid MSDs caused by erroneous work postures, however, more subjects are required to draw a definite conclusion.

## Introduction

Work-related musculoskeletal disorders (MSDs) are a major public health issue worldwide, causing lost QALYs (quality-adjusted life-year) and economical loss through work absences. Dentists are particularly exposed to the risk of MSDs because the profession itself implies repetitive motions, prolonged static postures, excessive exertion of small muscles, tight grip of instruments and raised arms position [1]. Several studies argue that, among oral healthcare providers, injuries of the neck, arms and lower back regions are the most debilitating and widely diagnosed [2, 3]. Even though a standard seated position that reduces strain on the locomotor system exists, the ‘ISO 11226 Ergonomic-Evaluation of static working postures standard’, many dentists choose to practice in other postures due to habit.

Thermal imaging provides information regarding local differences in skin surface temperature thus locating areas of increased temperature on the surface of the body, data that can indicate inflammation of the underlying muscle groups and thus strain or injury [4]. Muscle activity and strain is correlated with higher surface temperatures due to increased metabolic activity [2]. This technique can help diagnose and evaluate the severity of muscle strain caused by erroneous work posture and in turn also provide valuable information regarding what type of behavior reduces the risk of long term muscle strain and injury.

## Aim

The main objective of this study is to evaluate, using thermal imaging, the muscular strain experienced by oral healthcare professionals during work depending on their position, and to assess whether periodic stretching exercises have an impact on preventing MSDs.

## Material and Methods

We evaluated through comparison, using a thermal imaging camera, four dentists working in two different positions around the dental unit at different moments in time. The working positions for each subject were: Subjects 1 & 4 – the ISO 11226 Ergonomic seated position (the currently recommended standard) and Subject 2 and 3 – the flawed but widely used “9 o’clock” working posture (that puts extra strain on the right arm, exaggerated head-neck angle and forearms held too high). The ISO 11226 recommended working posture implies symmetrical postures – where all horizontal axes should be as close to parallel as possible, legs slightly apart (a 30-45º angle), the shank should be perpendicular on the floor, the upper part of the body should be perpendicular on the chair, any forward movements should be made without curving the spine, the head can bend 2025º, the arms should be kept close to the body while the forearms nearly horizontal (max. 25% raised)-the shank/thigh angle should be around115º and the soles should be completely resting on the floor [1].

**Figure 1:**
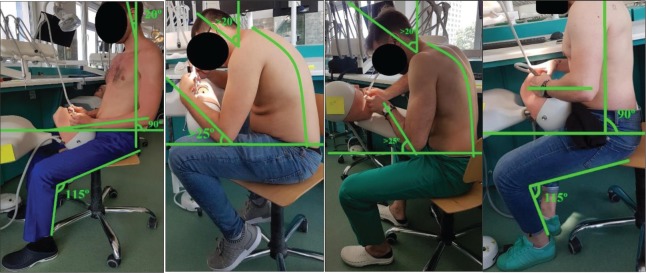
Working positions of subjects 1,2,3 & 4

The moments selected for imaging were as follows: T0 – initial moment, T1 – after 15 minutes of work related activity, T2 – after 30 minutes, and T3 – after stretching exercises.

The points of temperature measurement using the thermal camera on the subject’s body were the lumbar – labeled Bx1 (on thermography images), cervical – labeled Bx2, and right arm – labeled Bx3 areas, for each of the 4 subjects. The selected anatomical regions correspond to the trapezius, [5] triceps, and paravertebral muscles, [6] the ones most exposed to strain and injuries [1].

We performed thermal imaging scans of the subjects while working at all four chosen moments and evaluated the temperatures of all three anatomical areas for each subject. The hardware used was a FLIR B200 thermal imaging camera with a -20200ºC temperature range. Measurements were performed in a simulated dental office lab, at the Department of Dental Ergonomics of the University, under controlled environmental conditions (constant ambient temperature and humidity, no air circulation or cooling systems working at the moment of measurement).

## Results

**Figure 2:**
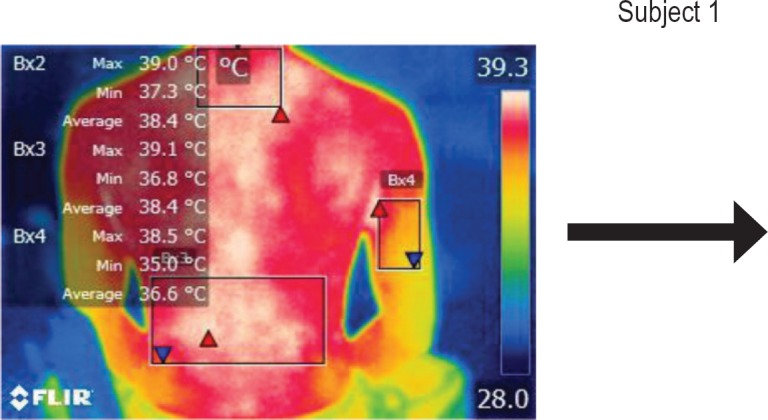
T0

**Figure 3:**
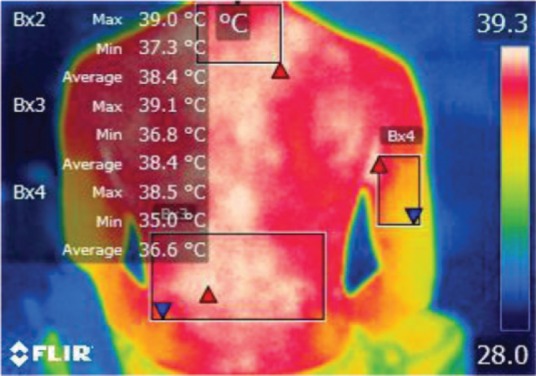
T1

**Figure 4:**
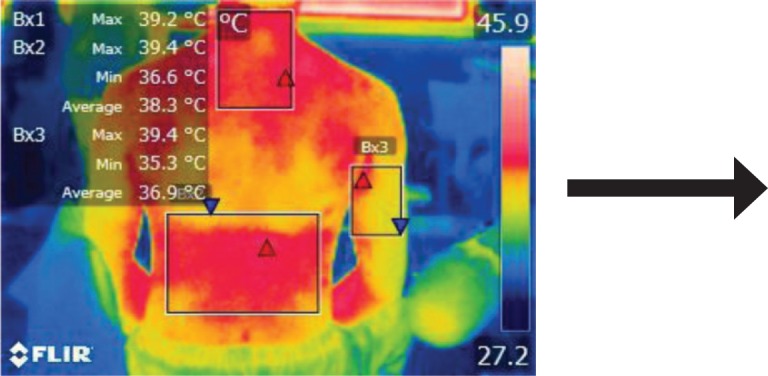
T2

**Figure 5:**
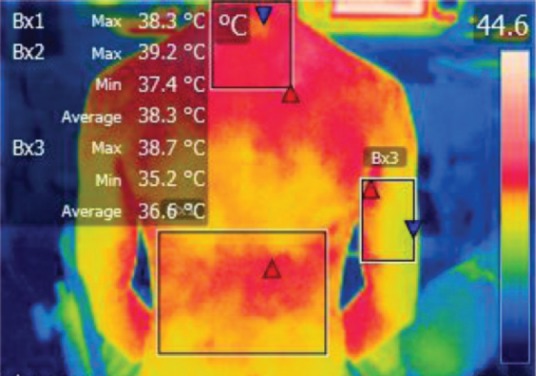
T3

**Figure 6:**
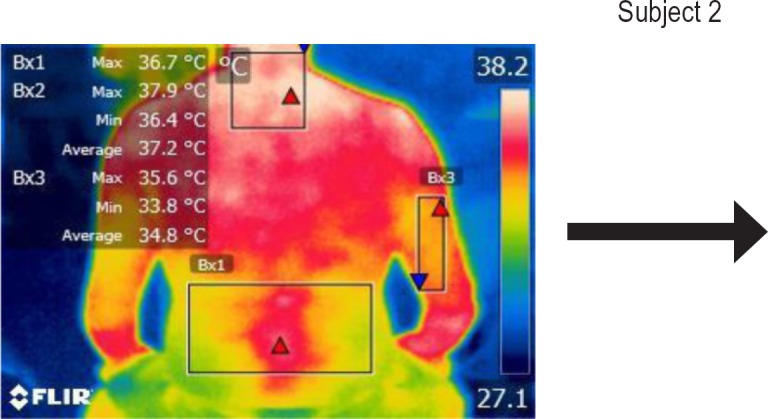
T0

**Figure 7:**
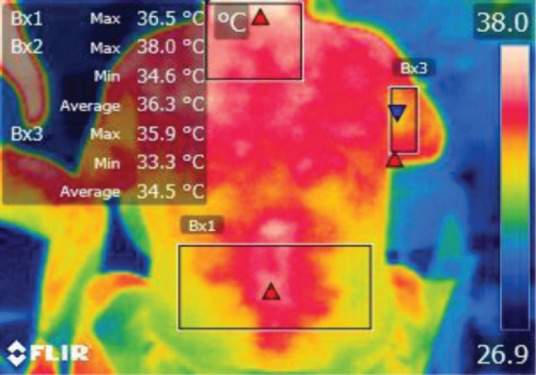
T1

**Figure 8:**
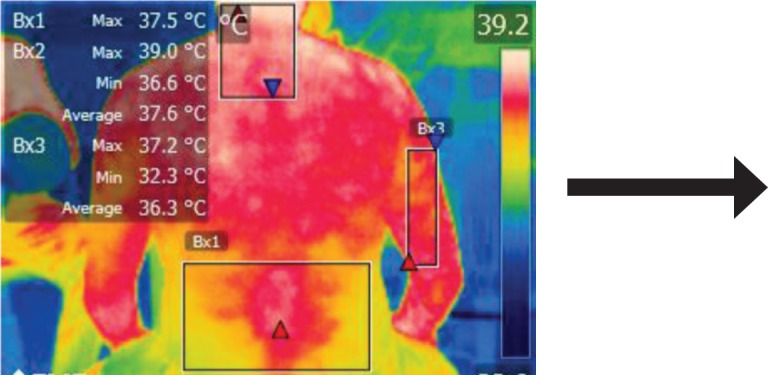
T2

**Figure 9:**
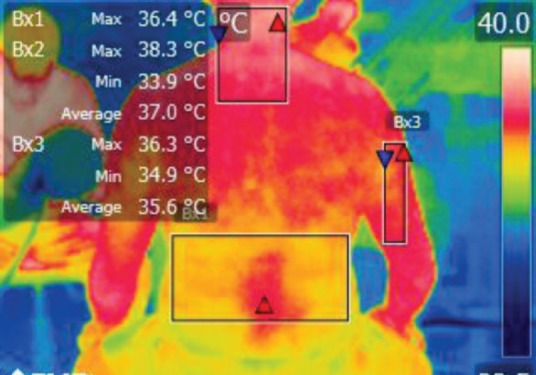
T3

**Figure 10:**
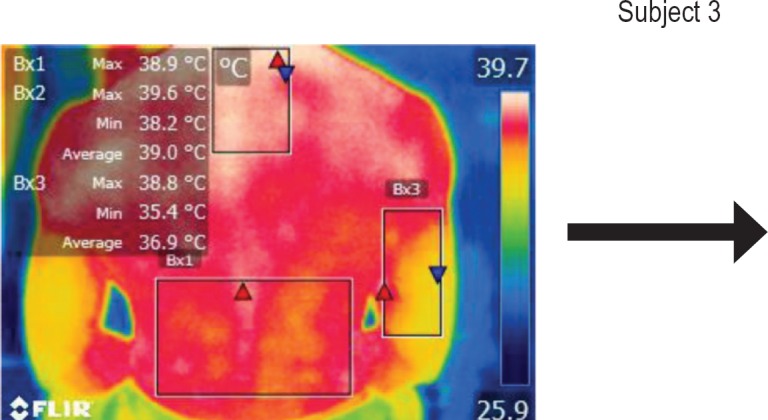
T0

**Figure 11:**
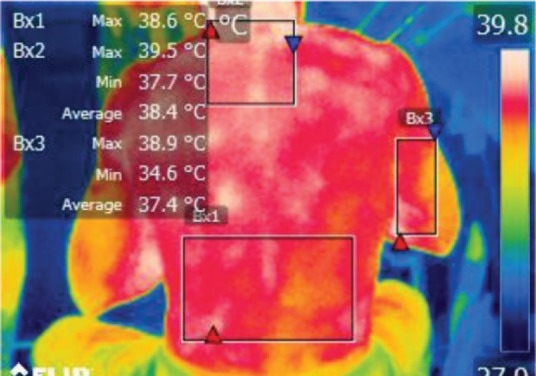
T1

**Figure 12:**
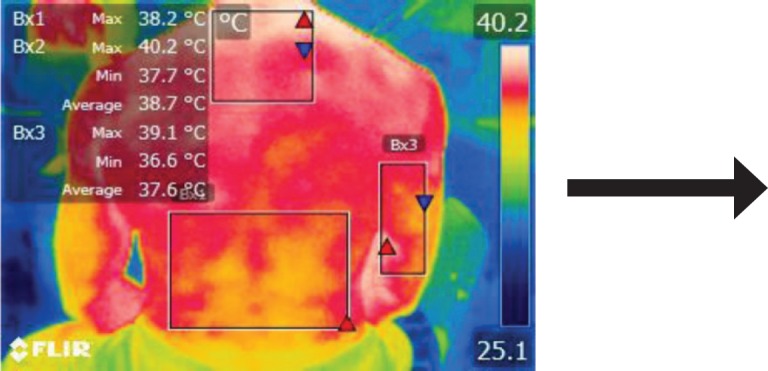
T2

**Figure 13:**
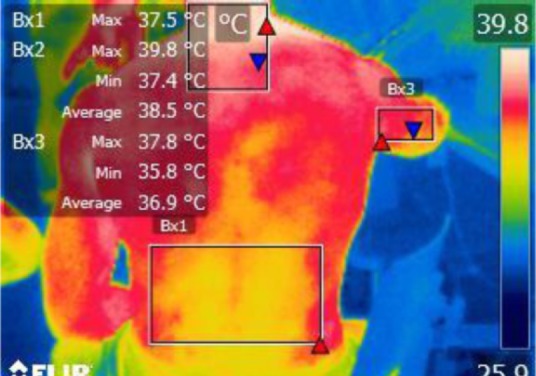
T3

**Figure 14:**
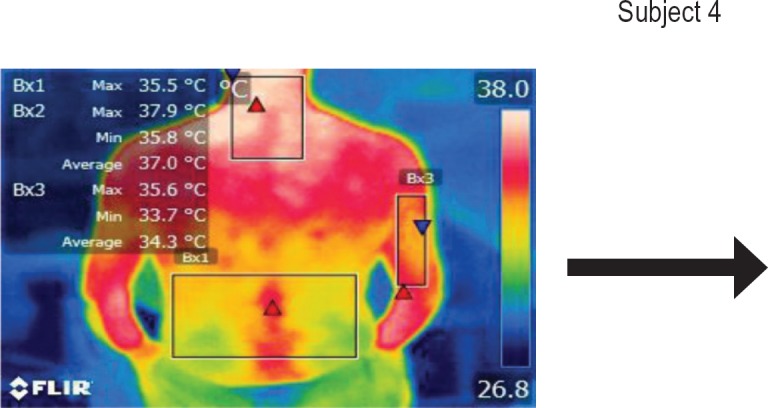
T0

**Figure 15:**
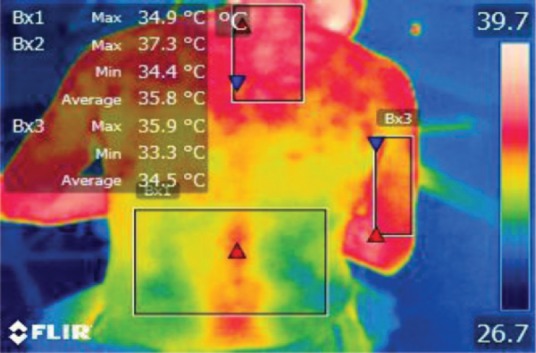
T1

**Figure 16:**
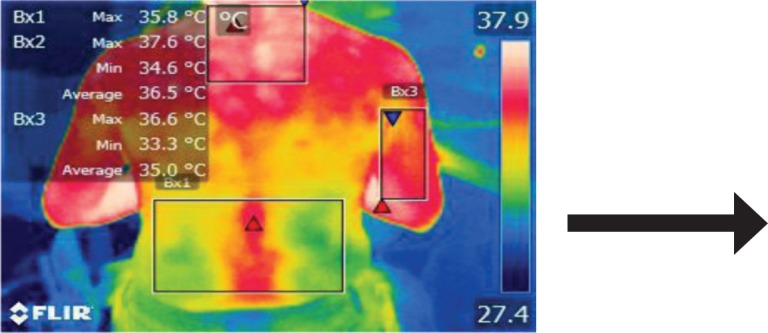
T2

**Figure 17:**
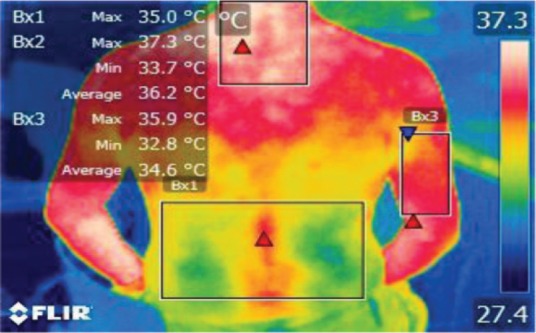
T3

## Discussions

Work related musculoskeletal disorders (WMSDs) are one of the leading causes of work absenteeism [7]. Studies have proven that thermal imaging can be used as a rapid, noninvasive and complementary diagnostic and evaluation method in many cases of musculoskeletal disorders [8]. Thermal imaging also possesses the advantage of having no risk for the subject (it does not encompass ionizing radiation or other physical or chemical hazards) and thus is a highly repeatable medical examination.

In this study we have tried to visualize, using skin surface temperature determinations via thermal imaging, the relationship between an increased surface temperature and strain/injuries of the underlying muscle groups, while also trying to highlight the positive effect of periodical stretching exercises translated through the lowering of these skin surface temperatures. However, the small number of subjects included in this study does represent a weak point and requires further work.

The inclusion and measuring of a greater number of subjects is required in order to properly standardize and organize a comprehensive screening protocol using thermal imaging for the diagnosis and staging of musculoskeletal disorders in oral healthcare professionals.

**Chart 1:**
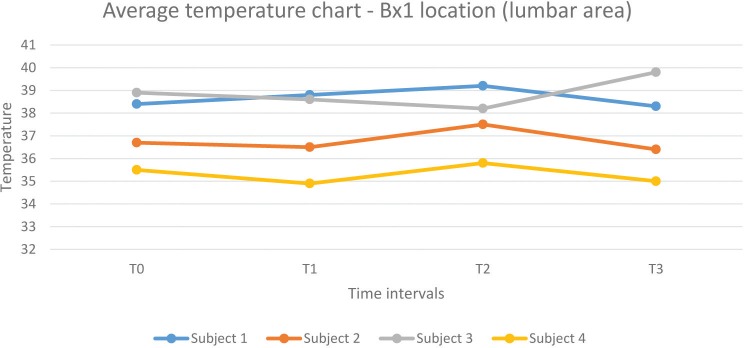


**Chart 2:**
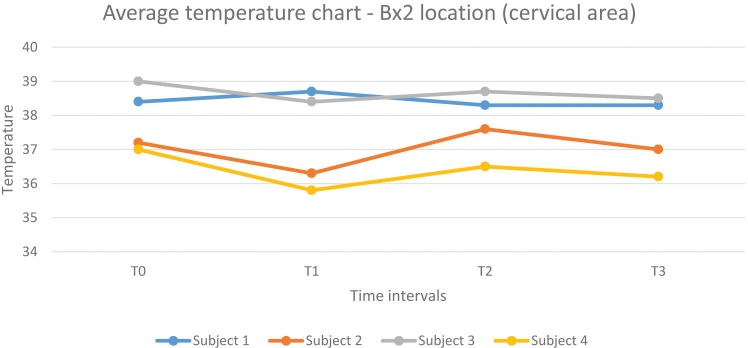


**Chart 3:**
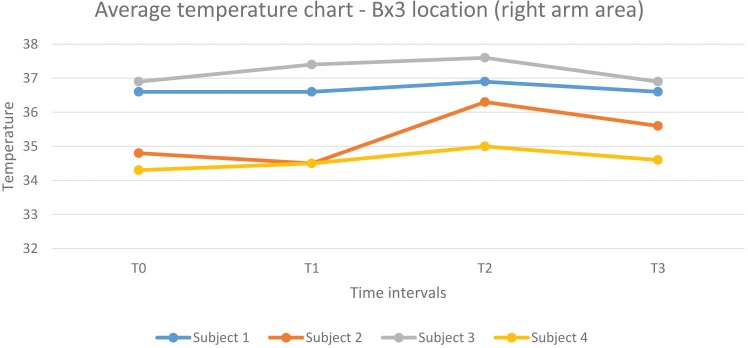


## Conclusions

1. Average temperatures of all three anatomical areas analyzed in all subjects seemed to be evenly distributed without any influence regarding the work position. In two subjects (one working in the correct position, the other in a flawed posture) the average temperature recorded have been consistently higher than in the other two.

2. Subjects experienced an increase in surface temperature from the T1 to the T2 time frame across all areas (the only exceptions being Subject 1 – Bx2 location and Subject 3 – Bx1 location), corresponding to an accentuation of muscle strain in the respective anatomical regions as time spent working in the incorrect position passed by.

3. Stretching exercises did provide a marginal decrease in surface temperature across all subjects and scanned areas – the exception being the Bx1 area in Subject 3.

## Conflict of Interest

The authors confirm that there are no conflicts of interest.
